# Non-Coding RNAs and the Development of Chemoresistance to Docetaxel in Prostate Cancer: Regulatory Interactions and Approaches Based on Machine Learning Methods

**DOI:** 10.3390/life13122304

**Published:** 2023-12-07

**Authors:** Elena Pudova, Anastasiya Kobelyatskaya, Marina Emelyanova, Anastasiya Snezhkina, Maria Fedorova, Vladislav Pavlov, Zulfiya Guvatova, Alexandra Dalina, Anna Kudryavtseva

**Affiliations:** 1 Engelhardt Institute of Molecular Biology, Russian Academy of Sciences, 119991 Moscow, Russia; 2 Russian Clinical Research Center for Gerontology, Pirogov Russian National Research Medical University, Ministry of Healthcare of the Russian Federation, 129226 Moscow, Russia

**Keywords:** prostate cancer, chemoresistance, docetaxel, microRNAs, lncRNAs, circRNAs, exosomes, machine learning, deep learning

## Abstract

Chemotherapy based on taxane-class drugs is the gold standard for treating advanced stages of various oncological diseases. However, despite the favorable response trends, most patients eventually develop resistance to this therapy. Drug resistance is the result of a combination of different events in the tumor cells under the influence of the drug, a comprehensive understanding of which has yet to be determined. In this review, we examine the role of the major classes of non-coding RNAs in the development of chemoresistance in the case of prostate cancer, one of the most common and socially significant types of cancer in men worldwide. We will focus on recent findings from experimental studies regarding the prognostic potential of the identified non-coding RNAs. Additionally, we will explore novel approaches based on machine learning to study these regulatory molecules, including their role in the development of drug resistance.

## 1. Introduction

Prostate cancer (PCa) is a socially significant cancer and is the second most common type of cancer in men worldwide [1]. Most patients with PCa have localized disease and are treated with radical prostatectomy and/or radiation therapy followed by androgen deprivation therapy. However, within 10 years after androgen deprivation in patients, in 10–20% of cases, the disease develops into a prognostically unfavorable form—castration-resistant prostate cancer (CRPC), which is characterized by a significant deterioration in the quality of life and high mortality of patients. The median overall survival of patients with CRPC is less than 2 years [2]. Chemotherapy based on taxane-class drugs, such as paclitaxel and docetaxel, is the gold standard for the first-line therapy in this patient category. Docetaxel is the most commonly used first-line chemotherapy drug for CRPC. However, despite its widespread use in therapy, most patients eventually develop resistance, which is one of the reasons for the ineffectiveness of chemotherapy in patients [3].

Drug resistance is conventionally divided into two main classes: primary (existing initially) and acquired [4]. Primary resistance is characterized by the presence of various factors in tumor cells prior to the action of the drug, whereas acquired resistance represents a stepwise and slower process involving various molecular genetic and epigenetic events in the presence of the drug [5]. The mechanisms underlying acquired drug resistance are quite complex and involve alterations in the regulation of numerous genes and different signaling pathways, which act independently or in combination with other factors to inhibit the function of taxanes in tumor cells. Several studies have identified a range of mechanisms involved in the development of taxane resistance, yet the full extent of the picture is still to be determined [6].

For a long time, non-coding RNAs were considered as by-products of transcription with little biological significance, in contrast to messenger RNAs (mRNAs). However, since the development of approaches such as high-throughput sequencing and in-depth bioinformatic analyses, new types of RNAs that do not encode proteins, collectively known as non-coding RNAs (ncRNAs), have been discovered. Currently, the most studied types of “classic ncRNAs” are microRNAs (miRNAs), long non-coding RNAs (lncRNAs), and circular RNAs (circRNAs). It has been shown that these molecules participate in many signaling cascades, regulate various physiological processes, and also play a role in diseases. Moreover, many ncRNAs have been identified as tumor suppressors/oncogenic factors in various types of cancer, including prostate cancer, and they may also participate in the development of drug resistance [7].

In addition to the regulatory role of ncRNAs in the context of drug resistance directly in tumor cells, the modulation of resistance development through tumor-derived exosomes has also been observed. Exosomes are spherical extracellular vesicles with a diameter of 40–150 nm containing various regulatory molecules (including ncRNAs) that have been found in various biological fluids. These vesicles mediate intercellular communication and perform important functions in tumor biology, such as inducing proliferation, angiogenesis, metastasis, and more. Furthermore, it has been shown that exosomes from chemosensitive/resistant tumor cells can significantly influence other tumor cells during chemotherapy by transferring specific regulatory molecules [8].

Considering the fact that the content of tumor-derived exosomes closely reflects the characteristics and metabolic status of their releasing cells, as well as the abundance, stability, and accessibility of exosomes in biological fluids, these vesicles represent a valuable source of biomarkers, including ncRNAs, for cancer diagnosis and the prediction of response to drug therapy [9,10,11].

Despite the existing experimentally confirmed data on the connections between ncRNAs and the development of drug resistance, their full role in this process remains unclear. Experimental research results can unveil new associations between ncRNAs and drug resistance. However, they present a challenging task due to significant time and financial investments. Addressing this issue can be achieved by employing computational methods that can predict potential connections between ncRNAs and drug resistance. In recent years, the use of machine learning methods has achieved a new level of forecasting for regulatory interactions between different molecules (such as DNA–RNA, RNA–microRNA, etc.) and associations between molecules and biological processes, including the correlation between ncRNAs and drugs [12]. Thus, machine learning methods can be successfully applied to predict potential connections between ncRNAs and drug resistance, allowing for in-depth and extensive exploration of the influence of these regulatory molecules on the development of drug resistance in tumor cells. In this review, we will examine the most prominent representatives of each class of ncRNAs, which recent studies have shown to play a significant role in the development of docetaxel resistance in PCa, and we will also explore novel approaches for studying ncRNAs in cancer using various machine learning-based tools.

## 2. MicroRNAs

MicroRNAs (miRNAs) are regulatory molecules with a length of 20–22 nucleotides that are formed from longer stem-loop structures and can bind to and inhibit mRNA. These molecules are transcribed as primary miRNAs (pri-miRNAs) and processed in the nucleus by the proteins Drosha and Dgcr8 into precursor miRNAs (pre-miRNAs). After export to the cytoplasm, pre-miRNAs are cleaved, forming a miRNA/miRNA duplex. Only one of the two miRNAs formed will exert its inhibitory function, while the other undergoes degradation. The total number of known human miRNAs is constantly expanding and currently includes 1917 precursors and 2654 mature molecules (miRBase, version 22.1).

**Figure 1:**
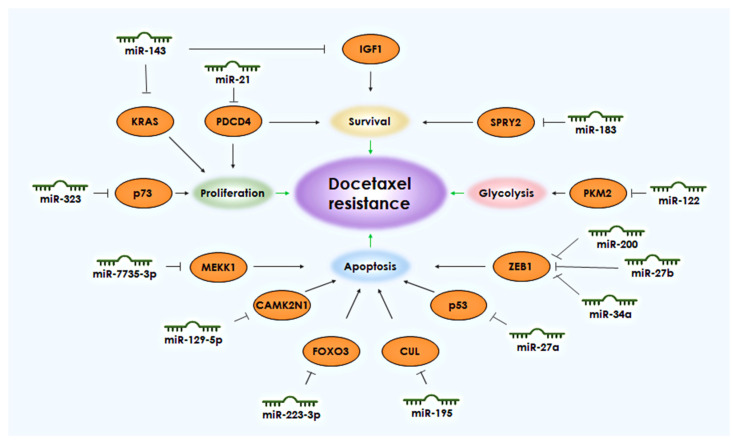
miRNAs and their targets associated with the development of resistance to docetaxel in PCa. The black arrows shows the connection between the target and the biological process. The green arrows indicate the connection of the process with the development of resistance to docetaxel.

Many miRNAs are often suppressed in drug-resistant cells, but under normal cellular conditions, they help regulate signaling pathways that promote cell survival. The ability to avoid programmed cell death, apoptosis, is one of the key characteristics of tumor cells that ensures their survival. Tumor cells, like normal cells, also possess proteins from the BCL-2 family that regulate apoptosis. These include Bad and Bax, which initiate cascades that activate apoptosis, and Bcl-2, Bcl-XL, and Mcl-1, which inhibit the apoptosis process [6]. Several microRNAs (miRNAs) have been demonstrated through various studies to regulate the development of docetaxel resistance in PCa cells by enhancing cell survival and inhibiting apoptosis. These miRNAs include miR-143, the miR-200 family, miR-21, miR-129-5p, miR-27a, miR-27b, miR-34a, miR-183, miR-195, miR-223-3p, miR-323, and miR-4735-3p (Figure 1, Table 1).

The downregulation of miR-143 can induce docetaxel resistance by enhancing the regulation of insulin-like growth factor-1 receptor (*IGF-1R*) and insulin receptor substrate 1 (*IRS1*), resulting in the activation of downstream signaling molecules such as VEGF (vascular endothelial growth factor A). Activation of the IGF-1R/IRS1/VEGF signaling cascade promotes the survival pathways of PCa cells and reduces sensitivity to docetaxel [13]. Furthermore, it has been found that miR-143 can regulate the *KRAS* (KRAS Proto-Oncogene, GTPase) gene, which is involved in the activation of the oncogenic MAPK/Ras pathway, and the overexpression of miR-143 can increase the sensitivity of cells to docetaxel [14].

Regarding the expression of the miR-200 family of regulatory molecules, a correlation has been found with participants involved in epithelial–mesenchymal transition such as E-cadherin and *ZEB1* (Zinc Finger E-Box Binding Homeobox 1). Decreased expression of miR-200 family members leads to increased expression of *ZEB1*, which negatively regulates E-cadherin. It was experimentally shown that the overexpression of miR-200 family members in PCa cells resulted in increased expression of E-cadherin and increased apoptosis induced by docetaxel [15]. Inhibition of epithelial–mesenchymal transition by suppressing the expression of the *ZEB1* gene has also been found in the context of docetaxel resistance in PCa cells due to the overexpression of miR-27b and miR-34a [16].

Similarly, miRNAs whose expression is normally at low levels can excessively stimulate cell survival pathways or inhibit pro-apoptotic factors. For example, it has been shown that miR-21 can inhibit programmed cell death 4 (*PDCD4*), leading to a resistant phenotype in PCa cells. Inhibiting miR-21 in this case increases the expression of *PDCD4* and restores sensitivity to docetaxel [17].

Regarding miR-129-5p, it has been shown that increased expression of this regulatory molecule leads to the suppression of the *CAMK2N1* (Calcium/Calmodulin Dependent Protein Kinase II Inhibitor 1) gene, resulting in the inhibition of apoptosis in PCa cells and the development of docetaxel resistance [18].

The most well-known tumor suppressor gene is *TP53* (Tumor Protein P53), and alterations in its expression or function are often associated with resistance to standard anti-cancer agents. In the case of PCa, it has been shown that exosomal miR-27a can induce resistance to cisplatin, docetaxel, and doxorubicin in recipient cells by degrading p53 mRNA, resulting in reduced expression of the negative regulator of the PI3k/Akt signaling pathway, *PTEN* (Phosphatase and Tensin Homolog). This leads to decreased dephosphorylation of PIP3, increased cell survival, and proliferation of tumor cells [19].

miR-183 also regulates cell survival pathways in PCa and contributes to docetaxel resistance. It has been shown that miR-183 regulates the expression of the tumor suppressor gene *SPRY2* (Sprouty RTK Signaling Antagonist 2), and its activation inhibits *SPRY2* expression, thereby promoting resistance to docetaxel [20].

The expression level of miR-195 is often decreased in docetaxel-resistant cells. It has been demonstrated that the *CUL* ( Cullin 3) gene, which is involved in the anti-apoptotic mechanism of docetaxel-resistant cells, is a direct target of miR-195. Thus, overexpression of miR-195 inhibits *CUL* gene expression and increases the sensitivity of PCa cells to docetaxel [21].

Regarding miR-223-3p, miR-323, and miR-4735-3p, their increased expression in PCa cells inhibits docetaxel-induced apoptosis. miR-223-3p directly targets the *FOXO3* (Forkhead Box O3) gene, miR-323 targets the tumor suppressor *p73*, while miR-4735-3p inhibits the expression of the *MEKK1* (Mitogen-Activated Protein Kinase Kinase Kinase 1; also known as *MAP3K1*) gene [22,23,24].

The expression of miR-122 has also been linked to the development of docetaxel resistance, but through another key process for the survival of tumor cells, glycolysis. It is known that a high level of *PKM2* (Pyruvate Kinase M1/2) expression is crucial for inducing glycolysis. However, it has been shown that miR-122 reduces the expression level of *PKM2* to inhibit glycolysis, which also leads to the reversal of resistance in tumor cells to docetaxel [25].

**Table 1:** The influence of microRNA expression on the resistance to docetaxel.

miR	Target	Process	Effect	Reference
miR-143	*KRAS*	proliferation, migration	inhibition	[14]
miR-143	*IGF1*	survival	inhibition	[13]
miR-200	*ZEB1*	apoptosis	inhibition	[15]
miR-21	*PDCD4*	proliferation, survival	induction	[17]
miR-122	*PKM2*	glycolysis	induction	[25]
miR-323	*p73*	proliferation	induction	[23]
miR-129-5p	*CAMK2N1*	apoptosis	induction	[18]
miR-27a	*P53*	apoptosis	induction	[19]
miR-27b	*ZEB1*	EMT, apoptosis	inhibition	[16]
miR-34a	*ZEB1*	EMT, apoptosis	inhibition	[16]
miR-183	*SPRY2*	survival	induction	[20]
miR-195	*CUL*	apoptosis	inhibition	[21]
miR-223-3p	*FOXO3*	apoptosis	induction	[22]
miR-4735-3p	*MEKK1*	apoptosis	induction	[24]

## 3. LncRNAs

Long non-coding RNAs (lncRNAs) are regulatory molecules that are more than 200 nucleotides long and can be transcribed from introns, exons, intergenic regions, or non-coding regions. These molecules can perform various functions, such as participating in transcription as factors, acting as “sponges” in protein–protein interactions, but their most interesting action lies in inhibiting miRNAs. Specific lncRNAs can act as competing endogenous RNAs (ceRNAs), which sequester or inhibit microRNAs involved in pro-apoptotic pathways [26]. The exact number of known lncRNA genes is constantly increasing, and currently, the number of registered lncRNAs has reached 100,000 [27]. There are now hundreds of thousands of cataloged lncRNAs and dozens of databases with carefully curated information, such as LncATLAS, LncBook, LNCipedia, lncRNAKB, and others [28].

**Figure 2:**
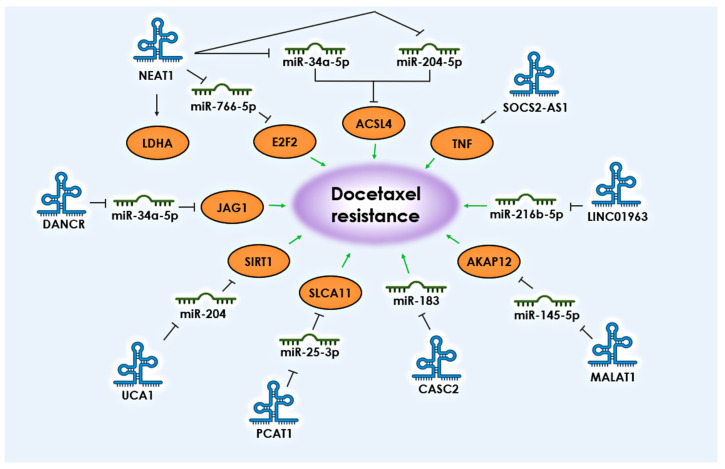
lncRNAs and their targets associated with the development of resistance to docetaxel in PCa. The black arrows shows the direction of communication between participants. Green arrows indicate the association with the development of docetaxel resistance.

The upregulation of key lncRNAs and the subsequent suppression of corresponding miRNAs is also associated with taxane resistance in PCa. Based on various studies, a number of lncRNAs have been identified whose interaction with miRNAs is associated with the development of docetaxel resistance in PCa*: NEAT1, UCA1, PCAT1, DANCR, CASC2, MALAT1*, and *LINC01963* (Figure 2, Table 2).

According to research findings, lncRNA *NEAT1* is considered a promising target for PCa treatment. Its increased expression leads to enhanced glycolysis by activating the *LDHA* (Lactate Dehydrogenase A) gene and suppressing T-cell immune surveillance [29]. Furthermore, *NEAT1* plays an important role in PCa oncogenesis by acting as a sponge for miRNA-766-5p, resulting in increased expression of the transcription factor *E2F3* [24]. High levels of *NEAT1* expression are also associated with docetaxel resistance in PCa, through the suppression of miR-34a-5p and miR-204-5p, leading to increased expression of the *ACSL4* (Acyl-CoA Synthetase Long Chain Family Member 4) gene, thereby promoting tumor progression and development of chemoresistance [30].

The activation of another lncRNA, *UCA1*, has also shown a connection with decreased expression of miR-204 and a positive correlation with increased expression of Sirt1, both of which regulate drug-induced apoptosis avoidance [31].

An important role in regulating the response to docetaxel in PCa has been found for lncRNA *PCAT1*, and there is increasing evidence of its involvement in PCa progression regulation [32]. It has been shown that the expression level of *PCAT1* increases under the influence of *TFAP2C* (Transcription Factor AP-2 Gamma) in PCa cells. As a result of *PCAT1* overexpression, the expression level of the *SLCA11* (Solute Carrier Family 4 Member 11) gene is increased by binding to miR-25-3p. This process is crucial for preventing the death of tumor cells from ferroptosis and developing resistance to docetaxel [32].

LncRNA *DANCR* is significantly activated in docetaxel-resistant PCa. Research has shown that *DANCR* suppresses the degradation of *JAG1* (Jagged Canonical Notch Ligand 1) induced by miR-34a-5p, thereby causing resistance to docetaxel [33]. Another lncRNA, *CASC2*, acts as a tumor suppressor in human malignancies, serving as a ceRNA for miR-183 and positively amplifying the expression of another tumor suppressor, *SPRY2*, a key antagonist of receptor tyrosine kinase signaling. The overexpression of *CASC2* and *SPRY2* can suppress the proliferation of PCa cells, promote their apoptosis, and increase sensitivity to docetaxel [34].

LncRNA *MALAT1* is currently the most well-characterized lncRNA, and its aberrant expression is observed in various types of cancer, including PCa [35]. It has been shown that *MALAT1* is involved in CRPC progression both in vivo and in vitro. Silencing *MALAT1* can inhibit tumor cell proliferation by arresting the cell cycle at the G0/G1 phase [36]. Additionally, it has been demonstrated that *MALAT1* enhances the expression of *AKAP12* (A-Kinase Anchoring Protein 12) gene by directly targeting miR-145-5p, promoting resistance to docetaxel [37].

There is also emerging evidence of another lncRNA, *LINC01963*, which has been associated with the development of docetaxel resistance in PCa. It has been found that overexpression of this lncRNA increases chemoresistance of PC3 cells to docetaxel by binding to miRNA-216b-5p [38].

In this section, we will also discuss the lncRNA that plays a role in the response to docetaxel independent of miRNAs-*SOCS2-AS1*. This lncRNA is an androgen-regulated regulatory molecule that is overexpressed in long-term androgen-deprived CRPC cells. *SOCS2-AS1* promotes androgen receptor signaling by suppressing its apoptotic target genes. Knockdown of *SOCS2-AS1* activates the TNF gene family and increases cell sensitivity to docetaxel, while overexpression of *SOCS2-AS1* induces resistance [39].

**Table 2:** The influence of lncRNAs expression on the resistance to docetaxel.

lncRNA	Target	Process	Effect	Reference
*NEAT1*	*LDHA*	glycolysis	induction	[29]
*NEAT1*	miR-766-5p	overexpression of transcription factor E2F3	induction	[30]
*NEAT1*	miR-34a-5p and miR-204-5p	increased expression of the *ACSL4* gene	induction	[31]
*UCA1*	miR-204	increased expression of Sirt1, avoidance of apoptosis	induction	[32]
*PCAT1*	miR-25-3p	increase in *SLCA11* gene expression, avoidance of ferroptosis	induction	[33]
*DANCR*	miR-34a-5p	increased expression of *JAG1* gene	induction	[34]
*CASC2*	miR-183	proliferation and apoptosis	inhibition	[20]
*MALAT1*	miR-145-5p	increased expression of *AKAP12* gene	induction	[37]
*LINC01963*	miR-216b-5p	-	induction	[38]
*SOCS2-AS1*	TNF family genes	androgen receptor signaling by suppressing its apoptotic target genes	induction	[39]

## 4. CircRNAs

Circular RNAs (circRNAs) represent a new class of non-coding single-stranded RNA molecules that are covalently linked to form a continuous closed loop and participate in the regulation of transcriptional and post-transcriptional gene expression [40]. Circular RNAs perform numerous unique and important biological functions: they act as traps for miRNAs or proteins, serve as scaffolds for circRNA–protein complexes, and recruit proteins to specific loci [40,41]. Additionally, some circRNAs can be translated into small unique peptides [42]. Research has shown that circRNAs promote tumor progression in various types of cancer by acting as RNA sponges and interacting with miRNAs, thereby enhancing gene expression [43].

CircRNAs are formed in circular transcripts through back-splicing of premature mRNAs, resulting in various types of circRNAs, such as exonic–intronic circRNAs (ElcircRNAs) (consisting of both exons and introns), circular intronic RNAs (formed by introns), exonic circRNAs (resulting from splicing of introns), and tRNA intronic circRNAs (formed by pre-tRNA splicing) [44]. To date, the total number of known circRNAs varies depending on the database. For example, the isoCirc database includes 107,147 full-length circRNA isoforms in 12 human tissues and one human cell line (HEK293), including 40,628 isoforms ≥ 500 nt in length [45]. The CircAtlas database contains 1,007,087 highly reliable circRNAs, with over 81.3% of them assembled into full-length sequences [46].

Based on the research, the crucial role of circRNAs in the development of chemoresistance in PCa has been highlighted, particularly regarding the molecules hsa_circ_0000735 and circFOXO3. The circular RNA hsa_circ_0000735 is activated in tissues and cells of docetaxel-resistant PCa. Functional analyses have shown that the suppression of hsa_circ_0000735 inhibits docetaxel resistance and suppresses tumor progression. Moreover, hsa_circ_0000735 can act as a sponge for miR-7, whose expression is decreased in docetaxel-resistant cells, thus promoting chemoresistance in PCa [47]. CircRNA circFOXO3 is one of the most studied circRNAs, and its inhibition has been shown to inhibit PCa progression and enhance docetaxel sensitivity by upregulating FOXO3 expression and repressing epithelial–mesenchymal transition of tumor cells [48].

## 5. Deep Learning-Based Tools to Study the Relationship between ncRNAs and Drug Resistance

Currently, one of the best machine learning approaches for solving prediction and classification problems in various studies is deep learning, a method based on neural networks that have many hidden layers and are based on the representation of biological neural networks. Over the past decade, deep learning has been successfully applied to various types of problems such as image recognition, speech, and language translation [49]. Deep learning is also widely used in bioinformatics, especially for working with RNA-Seq data, which is especially relevant for oncology research [50]. In this section, we want to highlight machine learning and deep learning tools for working with non-coding RNAs that deserve attention and can take research on the development of drug resistance in cancer to the next level.

First of all, it is worth noting that one of the key problems in research related to non-coding RNAs is their identification, which is difficult due to the similarity in length and sequence composition of protein-coding RNAs and ncRNAs. In the case of lncRNAs, tools such as lncRNAnet, lncADeep, and lncFinder have recently been proposed with promising results [51,52,53]. The identification of circRNAs from traditionally labeled mRNAs is also challenging due to the difficulty of analyzing experimental data and the relatively low expression of most circRNAs. Currently, the most accurate and preferred tool for identifying this class of regulatory molecules is circDeep [54]. Basic information about these algorithms and the key metrics used in these tools is presented in Table 3.

To analyze the assessment of the relationship between ncRNAs and the development of drug resistance, tools such as LRGCPND, GSLRDA, and DeepLDA are currently particularly interesting [12,55,56]. The basic information about the algorithms and the key metrics used in these tools is also presented in Table 3, but we will look at each of them in more detail.

LRGCPND is based on a graph computational convolutional neural network used to identify hidden relationships between ncRNAs and drug resistance through linear transition and residual prediction. This tool presents the relationship between ncRNAs and drug resistance in a bipartite plot and uses limited information to explore complex latent factors to predict boundaries. A bipartite graph is a structure whose vertices can be divided into two disjoint sets such that all edges connect a vertex in one set to a vertex in another set. The use of a bipartite graph in data analysis allows the integration of expression data for functional associations across many cancer types simultaneously [57]. A special feature of the LRGCPND tool is the fact that it first combines the potential features of neighboring nodes in each convolutional layer of the graph, and then transforms the information between layers using a linear function. Finally, LRGCPND combines the representations of each layer to complete the prediction. The authors showed that this tool can identify pairs of ncRNA–drug resistance associations, and in the specific case of cisplatin and paclitaxel, with an average AUC value of 0.8987 [55].

Another algorithm, GSLRDA, also predicts the connection between ncRNAs and drug resistance. In this tool, known associations between ncRNAs and drug resistance are modeled as a bipartite graph of ncRNAs and a drug, and GSLRDA uses a Light Graph Convolutional Network (lightGCN) to learn the embedding of ncRNAs and the drug from the bipartite ncRNA–drug graph. Additionally, GSLRDA employs various data augmentation methods to create diverse representations for ncRNAs and drug nodes and performs self-supervised learning, further improving the quality of learned ncRNAs and drug vector representations through contrastive learning between nodes. Finally, GSLRDA uses an inner product for predicting the connection between ncRNAs and drug resistance. The results of a large-scale analysis presented by the authors show that GSLRDA has an average AUC metric of 0.9101, which is higher than the other considered models on the data sets used [56].

Finally, it is worth mentioning DeepLDA, a deep learning-based computational model that uses deep neural networks and a graph attention engine to learn mRNA and drug embeddings to predict potential links between mRNAs and drug resistance. This model first uses known association elements to generate mRNA–drug similarity networks. Deep graph neural networks are then used to pre-train mRNA and drug properties, which are ultimately fed into a graph attention network to learn mRNA and drug embeddings to predict potential association pairs. The experimental results based on the NoncoRNA and ncDR databases have shown that the DeepLDA model currently outperforms other machine learning methods in predicting mRNA–drug resistance pairs on the data sets used with an AUC value of up to 0.8889 [12].

## 6. Discussion and Conclusions

Prostate cancer is one of the most common oncological diseases among men worldwide, and for treating advanced stages of the disease, chemotherapy based on taxanes is used as the “gold standard.” Despite significant progress in molecular oncology over the past decades, the development of drug resistance in patients remains one of the most pressing issues in modern oncology. The mechanisms underlying drug resistance in cancer are complex and involve changes in the regulation of numerous signaling pathways, although the complete picture of their interactions is yet to be determined.

In this review, we discussed the class of non-coding RNAs—regulatory molecules that play a key role in various cellular processes and diseases, including cancer. With the rapid development of high-throughput sequencing methods, it has been discovered that a large number of non-coding RNAs are aberrantly expressed in various tumor tissues and cell lines. While the dysregulation of non-coding RNAs can contribute to the emergence of cancer traits as oncogenes or counteract them as tumor suppressors, the mechanisms underlying these events are not fully understood. In recent years, increasing evidence has emerged regarding the important role of these molecules in the development of drug resistance in various types of cancer, including prostate cancer, by influencing multiple signaling pathways. This knowledge may be successfully applied in the future for the development of new promising therapeutic approaches.

We discussed the major non-coding RNAs that recent studies have shown to be closely associated with the development of docetaxel resistance in prostate cancer, including miRNAs (miR-143, -200, -21, -122, -323, -129-5p, -27a/b, -34a, -183, -195, -223-3p, -4735-3p), lncRNAs (*NEAT1, UCA1, PCAT1, DANCR, CASC2, MALAT1, LINC01963, SOCS2-AS1*), and circRNAs (hsa_circ_0000735 and circFOXO3).

The identification of non-coding RNAs and the elucidation of their relationship with diseases not only contribute to our understanding of the mechanisms of disease development but also provide new ideas and solutions for their diagnosis, treatment, and prognosis. Currently, research on predicting associations between non-coding RNAs and diseases is gaining more attention, accompanied by an increasing number of prediction methods based on machine learning. We also discussed modern computational tools based on machine learning and deep learning methods for the identification of non-coding RNAs from transcriptomic data, such as lncRNAnet, lncADeep, lncFinder, and circDeep. These tools aid in the identification of non-coding RNAs from transcriptomic data and in predicting potential links between non-coding RNAs and drug resistance in cancer, such as LRGCPND, GSLRDA, and DeepLDA.

## Figures and Tables

**Table 3:** Machine learning models for identifying ncRNAs and analyzing associations with the development of drug resistance.

Model	Application	Structure	Metrics	Reference
lncRNAnet	lncRNAs identification	convolutional neural network and recurrent neural network	Sensitivity, Specificity, Accuracy, F1 score, and AUC	[51]
lncADeep	lncRNAs identification	deep belief network	Sensitivity, Specificity, Accuracy, F1 score, Matthew’s correlation coefficient, and AUC	[52]
lncFinder	lncRNAs identification	random forest, support vector machine, logistic regression, extreme learning, machine and deep learning	Sensitivity, Specificity, Accuracy, and F1 score	[53]
circDeep	circRNAs identification	asymmetric convolution neural network и recurrent neural network	Accuracy, Matthew’s correlation coefficient, and F1 score	[54]
LRGCPND	ncRNAs and drug resistance association	graph convolution network	AUC, AUPR, Accuracy, Precision, Recall, F1 score	[55]
GSLRDA	ncRNAs and drug resistance association	bipartite graph, light graph convolutional network	AUC, F1 score	[56]
DeepLDA	lncRNAs and drug resistance association	graph neural network and graph attention mechanism	AUC, AUPR, F1 score, and Matthew’s correlation coefficient	[12]

## Data Availability

Not applicable.
